# Current progress in *Striga* management

**DOI:** 10.1093/plphys/kiab040

**Published:** 2021-02-05

**Authors:** Muhammad Jamil, Boubacar A Kountche, Salim Al-Babili

**Affiliations:** Division of Biological and Environmental Sciences and Engineering, the BioActives Lab, King Abdullah University of Science and Technology, Thuwal 23955-6900, Saudi Arabia

## Abstract

The Striga, particularly S. he rmonthica, problem has become a major threat to food security, exacerbating hunger and poverty in many African countries. A number of Striga control strategies have been proposed and tested during the past decade, however, further research efforts are still needed to provide sustainable and effective solutions to the Striga problem. In this paper, we provide an update on the recent progress and the approaches used in Striga management, and highlight emerging opportunities for developing new technologies to control this enigmatic parasite.

## Teaser

Recent progress and the approaches used in Striga management are summarized.

**Advances**

- The recently established Striga control technologies, such as push-pull, toothpick, and imidazolinone seed dressing have opened up new opportunities for smallholder farmers to overcome this parasite.
- The development of low-cost and efficient germination stimulants together with an application protocol for rain-fed agriculture has made the suicidal germination strategy a realistic approach.
- Molecular elucidation of strigolactone biosynthesis and perception has led to the development of new chemicals that disrupt the communication between Striga and its hosts.

## Background

*Striga* species of the Orobanchaceae family are obligate root parasites that infest staple crops in sub-Saharan Africa (SSA), Middle East, and parts of Asia (Tank et al., 2006; Parker, 2012). *Striga hermonthica*, *Striga asiatica*, and *Striga gesnerioides* are the most economically important parasitic plants among the 42 known *Striga* species. The three species differ in their host specificity. *Striga hermonthica* and *S asiatica* parasitize on cereals and sugarcane (*Saccharum officinarum*; Parker, 2009; Pennisi, 2010), while cowpea (*Vigna unguiculata*) is the main host of *S. gesnerioides* (Ohlson and Timko, 2020). The *Striga*, particularly *S. hermonthica*, problem has become a major threat to food security, exacerbating hunger, and poverty in many African countries (Pennisi, 2010; Khan et al., 2014). Although consequences are difficult to measure, a few estimates have indicated that *Striga* is affecting the life of more than 300 million people in Africa and causing enormous yield losses with a value ranging from 7 to 10 billion US$ annually (Emechebe et al., 2004; Gressel et al., 2004; Ejeta, 2007; Scholes and Press, 2008; Rodenburg et al., 2010). In heavily infested regions, farmers have been forced to abandon cereal cultivation and to switch to other less important crops (Atera et al., 2012a). The severity of *Striga* depends upon degree of infestation, seed viability, ecotypes, virulence, host crop susceptibility, climatic/edaphic factors, and cultural practices (Rodenburg et al., 2016).

*Striga* species are among the hardest parasitic plants to control (Berner et al., 1995; Nickrent and Musselman, 2004). Adaptability of *Striga* to a wide range of hosts and environmental conditions has made it one of the most widespread and successful parasitic plants (Mohamed et al., 2006). In addition, long-term management of *Striga* is hampered by the tremendous number and longevity of seeds, vast genetic variability, complex life cycle, and subterranean nature of damage (Joel, 2000; Huang et al., 2012a, b). Indeed, it is estimated that about 900,000 *S. hermonthica* plants can emerge from one hectare of infested sorghum field, which can add about 4.5 × 10^10^ seeds in one growing cycle (Bebawi et al., 1984). Very tiny (0.3 nm × 0.15 nm) and light (4–7 μg) *Striga* seeds are easily dispersible in nearby fields through wind, animals, and agricultural tools, thereby gradually enriching seed reserve in the soil (Ejeta, 2007). The seeds remain dormant for a long period and germinate only after exposure to hot and humid conditions followed by perception of host derived germination stimulants, mainly strigolactones (SLs; Yoneyama et al., 2010; Joel and Bar, 2013; Al-Babili and Bouwmeester, 2015). Following germination, *Striga* radicle grows toward host roots. The perception of host-derived haustorium-inducing factors (HIFs), such as 2,6-dimethoxy-1,4-benzoquinone, prevents further growth of the radicle and induces cell expansion and division, and proliferation of hair cells at its tip, forming a haustorium, a special invasive organ that penetrates host roots to enable siphoning off water, minerals, and nutrients. The vital role of the haustorium provides a rarely exploited option for controlling *Striga* by breeding varieties with low HIF release or developing compounds that specifically inhibit haustorium formation (Cechin and Press, 1993; Shen et al., 2006; Yoshida and Shirasu, 2012; Yoshida et al., 2016; Goyet et al., 2019).

**Figure 1:**
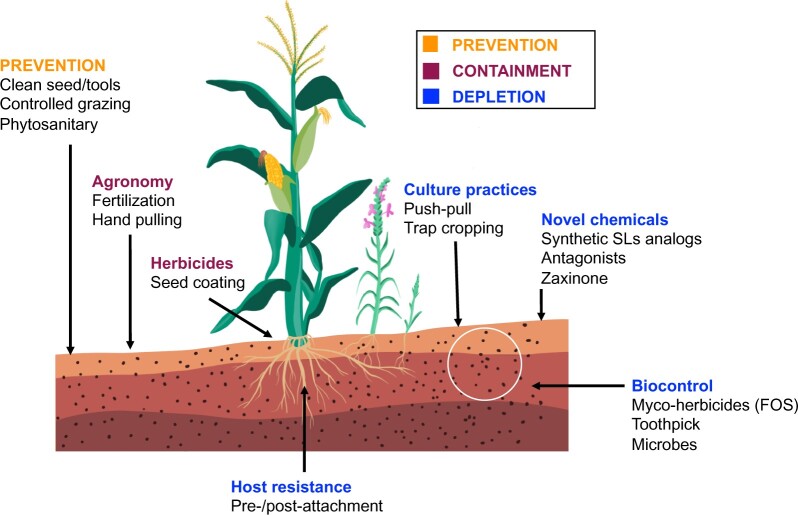
List of complementary approaches for *Striga* control. Methods depicted are used either for prevention and containment of *Striga* infestation, or for depletion of accumulated seed banks.

Due to the persistence and severity of the *Striga* problem, there has been an extensive effort to develop simple, easy, and effective control strategies that can be employed either alone or integrated with existing approaches (Figure 1). In the past decade, intensive research on the interaction of *Striga* with its host at molecular level has opened up opportunities to develop new management strategies. For sustainable *Striga* management, any control method should target at least one of the following goals (Figure 1): (1) PREVENTION: avoiding seed dispersal, for instance, by using clean crop seeds, tools, and fodder, controlling animal grazing or applying phytosanitary/quarantine measures; (2) CONTAINMENT: limiting new seed production by planting resistant (pre- and post-attachment, see below) varieties, using fertilizers, applying herbicides, employing a number of agronomic practices, such as hand weeding, deep sowing, burning, fallowing and soil solarization, and the application of chemicals that reduce the release of germination stimulants by the host; and (3) REDUCTION: reducing *Striga* seed bank accumulated in infested soils by employing cultural practices, such as trap cropping, inter cropping, and crop rotations, employing microbial agents that impact *Striga* and/or *Striga*/host interaction, applying synthetic germination stimulants in host’s absence, and by developing specific inhibitors that block germination of preconditioned seeds and decrease their viability. Reduction of seed bank can be also achieved by planting resistant varieties that stimulate *Striga* seed germination, but withstand the parasitic attack (post-attachment resistance, see below). In this article, we provide an update on the recent progress and the approaches used in *Striga* management, and highlight emerging opportunities for developing new technologies to control this enigmatic parasite.

### Host resistance

Deployment of resistant varieties is generally considered as the most economical, practical, and suitable long-term approach for controlling *Striga* (Hearne, 2009; Mandumbu et al., 2019). There are two main types of host resistance, that is, pre-attachment resistance resulting from SL profiles with low seed germinating activity, and post-attachment resistance that is based on hypersensitive response (HR)/incompatible response (IR). The recent identification of genes mediating the synthesis of different SLs opens up the possibility of modifying the SL profile of host plants by using genetic engineering and gene editing tools and, hence, increasing their resistance (Wakabayashi et al., 2019, 2020). Genetic resistance can be employed independently or as a central element of an integrated *Striga* management (ISM). Here, we present the latest knowledge on *Striga* resistance in maize (*Zea mays*), sorghum (*Sorghum bicolor*), pearl millet (*Pennisetum glaucum*), rice (*Oryza sativa*), and cowpea (*V. unguiculata*).

Maize has surpassed the traditional cereals in SSA, with the highest cultivation area of about 39 million ha in 2018 (FAOSTAT, 2018). Considering the importance of this crop, significant progress has been achieved in identifying a number of *Striga* resistant maize varieties and hybrids with different types of resistance (Akaogu et al., 2013, 2019; Badu-Apraku et al., 2019; Menkir and Meseka, 2019). For instance, Amusan et al. (2008) identified ZD05 a resistant maize inbred line that exhibits very low *Striga* attachments and high mortality of attached parasites, compared with the susceptible inbred line 5057. This resistance in ZD05 has been attributed to multilevel post-attachment barriers, particularly physiological or biochemical incompatibility to parasite growth and development. It is likely that this resistance is controlled by different genes that can be employed for durable and stable resistance. Similarly, Nyakurwa et al. (2018) compared quality protein maize (QPM) with non-QPM genotypes for *S. asiatica* resistance/tolerance under field/pot and agar gel assay conditions. They found several QPM genotypes with considerable levels of tolerance (i.e. no impairment of growth and yield despite of infestation), although they showed susceptibility similar to that of other genotypes in agar gel assays. However, the mechanisms underlying the tolerance of QPM genotypes remain elusive. In a similar study, Midega et al. (2016) evaluated six landraces for *Striga* infection under pot and natural field infestation and showed low *Striga* emergence, relative to hybrids.

In addition to investigating resistance in the field, a number of pre-attachment resistance studies based on SL analysis have been conducted in the past few years. For instance, Karaya et al. (2012) screened a collection of 420 maize landraces, populations, and inbred lines and identified several landraces with low *Striga* germinating activity and reduced release of the highly active strigol, however, without further investigation of responsible genetic factors. Later, the effect of SL composition on *Striga*/maize interaction was further demonstrated by comparing the two cultivars Pioneer 3253 (*Striga*-susceptible) and KSTP94 (resistant), which showed that 5-deoxystrigol was exclusively released by the susceptible variety, while sorgomol was the main SL in root exudates of the resistant one (Yoneyama et al., 2015). Interestingly, the two varieties were indistinguishable with respect to arbuscular mycorrhizal (AM) symbiosis, indicating that the effect of SL composition on the interaction with AM fungi is different from that on *Striga* attack (Yoneyama et al., 2015). Furthermore, a study of *S. hermonthica* infestation using rhizotron unraveled the presence of post-attachment resistance in the aforementioned cultivar KSTP94, demonstrated by lower number of *Striga* attachments and biomass in comparison to the susceptible maize inbred line CML144 (Mutinda et al., 2018). Similarly, Gasura et al. (2019) evaluated 30 maize inbred lines for *S. asiatica* resistance in pots and using agar gel assays, and identified seven inbred lines with low germination stimulant production and decreased root attachment and emergence under pot conditions, while four inbred lines exhibited low *Striga* attachment.

Recently, a genome-wide association study for *S. hermonthica* resistance in maize identified significant loci on chromosomes 3, 9, and 10, which are related to plant defense. A total of 24 single nucleotide polymorphisms (SNPs; under *Striga* infested conditions) and 11 SNPs (under *Striga*-free conditions) showed significant association with grain yield and number of ears per plant. The identified loci and candidate genes (*GRMZM2G060216*, *GRMZM2G057243*, and *GRMZM2G164743*) could be excellent breeding source for the development of *Striga*-resistant maize genotypes through marker-assisted selection (MAS) in SSA (Adewale et al., 2020). Moreover, as mentioned above, the ability of cultivars, such as KSTP94, to resist *Striga* infestation at pre- and post-attachment stages makes them a very suitable genetic source for resistance breeding. In addition, the identification of genetic factors underlying the resistance will allow stacking them by using genetic engineering/genome editing technologies to generate highly resistant varieties to *Striga* infestation.

Sorghum is the second most important cereal crop after maize in SSA. It was grown on about 30 million ha in 2018 (FAOSTAT, 2018). In the past two decades, significant progress has been made with the identification and characterization of quantitative trait loci (QTLs) associated with *Striga* resistance in this cereal. Initially, Haussmann et al. (2004) detected QTLs in recombinant inbred lines derived from the cross between IS9830 and N13. By deploying MAS techniques, some QTLs have been transferred into elite sorghum varieties, leading to the development of sorghum cultivars with improved resistance to *Striga* (Ejeta, 2007). Moreover, by using 328 recombinant inbred lines, derived from a cross between SRN39 (low germination stimulant) and Shanqui Red (high germination stimulant) sorghum, Satish et al. (2012) fine mapped *LOW GERMINATION STIMULANT 1* (*LGS1*) locus to a 400-kb region on chromosome 5. Interestingly, it was later demonstrated that mutations in *LGS1* (*lgs1-1* to *lgs1-5* mutants) lead to a change in the stereochemistry of released SLs, replacing the dominant SL 5-deoxystrigol by the less active orobanchol (Gobena et al., 2017). These findings were confirmed by Mohemed et al. (2018) who showed that sorghum genotypes with high release of 5-deoxystrigol are more susceptible to *Striga*, compared to orobanchol releasing genotypes. More importantly, *LGS1*-mediated (loss-of-function) resistance was further characterized in various sorghum landraces with respect to *S. hermonthica* diversity and geographic distribution (Bellis et al., 2020). These authors reported that *LGS1* loss-of-function mutations are adaptive and widely distributed among African landraces across a large region of highly *Striga* infestation in Africa. Using gene-edited sorghum lines, it has been further shown that the degree of *LGS1*-mediated resistance depends on parasite genotype and abiotic environment (Bellis et al., 2020). In addition to the well-characterized pre-attachment mechanism, few reports documented the presence of strong HR or incompatibility-based post-attachment resistance (Mohamed et al., 2010a, 2010b; Mbuvi et al., 2017).

Pearl millet was cultivated on around 22 million ha during 2018, making it the third most important cereal after maize and sorghum in SSA (FAOSTAT, 2018). However, with paucity of reliable and adapted resistance donor sources, research and breeding for *Striga* resistance in pearl millet is still challenging, compared with other cereals (Wilson et al., 2000, 2004; Kountche et al., 2013; Sattler et al., 2018). Although several studies have been conducted to characterize mechanisms and inheritance of resistance to *Striga* in other cereals (Amusan et al., 2008; Cissoko et al., 2011; Jamil et al, 2011a; Satish et al., 2012), knowledge on individual resistance mechanisms, their genetic and physiological basis are still lacking in pearl millet. To fill this gap and by deploying a genotyping-by-sequencing approach, a genetic map of SNP markers together with single sequence repeats was constructed using a segregating population derived from a cross between a wild relative, resistant to *Striga* (Wilson et al., 2004) and a cultivated-susceptible pearl millet parent (Moumouni et al., 2015). The availability of such genomic resources will help in identifying and mapping QTLs associated with *Striga* resistance, which can be deployed in a MAS. Also, using conventional field based breeding, significant progress has been made with the identification of six varieties (M141, M239, M029, M197, M017, and KBH), showing higher yield and less *Striga* susceptibility (Kountche et al., 2013). The response to five cycles of phenotypic recurrent selection for *Striga* resistance was evaluated in a diversified pearl millet gene pool developed from these varieties (Kountche et al., 2013). The authors also reported the development of the first *Striga*-resistant experimental varieties (Kountche et al., 2013).

Rice was grown in 2018 on about 14 million ha in SSA (FAOSTAT, 2018). Both, pre- and post-attachment resistances were detected in this cereal. The Nipponbare rice cultivar shows strong post-attachment resistance to *S. hermonthica*, likely because of its incapability to build xylem–xylem connections with the parasite (Gurney et al., 2006). To shed light on the genes underlying this resistance, the response of Nipponbare and the susceptible cultivar IAC 165 was investigated using gene expression profiling. This study unraveled an association of the induction of defense genes with resistance, and of that of genes involved in nutrient transport, amino acid metabolism and abiotic stress response with susceptibility (Swarbrick et al., 2008). Genes induced in the resistant cultivar include three with unknown function, which are localized within a major resistance QTL on chromosome 12 and might have a significant contribution to the resistance (Swarbrick et al., 2008). In another study, three *Striga* resistance QTLs were detected in Koshihikari–Kasalath backcross inbred lines. A QTL of major effect was verified and narrowed down and could be, therefore, a good target for MAS (Swarbrick et al., 2009). *Striga* attack on the resistant rice cultivar Nipponbare is accompanied by an accumulation of lignin, guaiacyl, and syringyl at the site of *Striga* infection and by induction of phenylpropanoid pathway genes. The role of lignification in Nipponbare post-attachment resistance was demonstrated through manipulation of genes regulating lignin composition, which led to *Striga* susceptibility in this cultivar (Mutuku et al., 2019). Besides this structural barrier, post-attachment resistance also depends on a change in the level of defense hormones. Transcriptome analysis of infested roots indicated an involvement of the plant hormones jasmonic acid (JA) and salicylic acid (SA) in *Striga* post-attachment resistance. Mutant analysis confirmed the role of JA, but not that of salicylic acid. However, *WRKY45* knockdown—a regulator of the SA/benzothiadiazole-mediated defense response—can lead to *Striga* susceptibility. This phenotype (susceptibility) could be rescued by exogenous JA application, indicating that WRKY45 contributes to *Striga* defense by modulating the interaction between JA and SA and positively regulating SA/benzothiadiazole and JA pathways (Mutuku et al., 2015).

A screen for pre-attachment resistance unraveled NEw RICe for Africa (NERICA) cultivars, such as NERICA1, and their parent CG14, which release low amounts of SLs (Jamil et al., 2011a). In another study, rice high tillering cultivars, for example, Super Basmati or TN1, showed low SL production and *Striga* infection, in contrast to low tillering rice varieties, for example, IAC-165 or IAC-1246 (Jamil et al., 2012a). Interestingly, Cissoko et al. (2011) reported that the above-mentioned NERICA cultivars also exhibited post-attachment resistance to *S. hermonthica* and *S. asiatica*, caused by incompatibility response or lack of xylem–xylem connections to rice endodermis. Both resistance studies were further validated and confirmed under field conditions (Atera et al., 2012b; Rodenburg et al., 2015, 2017). Similarly, the upland rice variety Umgar was characterized by pre- and post-attachment resistance to *S. hermonthica* under lab, pot, and field conditions (Samejima et al., 2016a).

Cowpea is an important vegetable and food legume in many African countries but its yield is severely affected by *Striga gesnerioides* (Parker, 2009). About seven distinct races of *S. gesnerioides* (SG1–SG6 and SG4z) were classified based on their genetics and parasitism on cowpea in West Africa (Botanga and Timko, 2005; Botanga and Timko 2007). Cowpea resistance to *S. gesnerioides* is conferred by single dominant genes in a race-specific manner (Timko and Singh, 2008; Timko et al., 2012). In addition, many cowpea landraces and local accessions possess post-attachment resistance that is based on HR at the site of attachment (Timko and Singh, 2008). Indeed, Li and Timko (2009) identified and characterized *RSG3‐301* from the cowpea cultivar B301, which is involved in *S. gesnerioides* resistance. Silencing of *RSG3-301* in B301 plants caused susceptibility to *S. gesnerioides* race SG3 due to reduced HR. These findings led to the conclusion that the race‐specific *Striga* resistance in cowpea is likely an effector‐triggered immunity that activates intracellular NLR proteins (RSG3-301) upon the recognition of pathogen/parasite effectors. Supporting the race-specific interactions in *S. gesneriodes*–cowpea associations, the cultivar B301 was, however, susceptible to the *S. gesnerioides* race SG4z producing a small soluble effector protein at high amounts in haustoria, which is transferred to the host root (Huang et al., 2012a, b). This protein can suppress the host innate immunity by binding to a host BTB‐BACK domain‐containing ubiquitin E3 ligase homolog POB1 (POZ/BTB containing protein 1). Overexpression of *VuPOB1* led to a reduction of SG4z parasitism due to increased HR, while its silencing caused susceptibility, suggesting that VuPOB1 might be a positive regulator of the HR response (Su et al., 2020). As mentioned above, *S. gesnerioides* races and their distribution are dynamic systems, influenced by genetic drift and gene flow. Ohlson and Timko (2020) recently investigated *S. gesnerioides* diversity and known sources of resistance in cowpea. They collected 58 unique *S. gesnerioides* populations from 9 West African countries and screened 7 cowpea lines for resistance. Results obtained showed that none of the cowpea lines was resistant to all *S. gesnerioides* populations and that there is no *S. gesnerioides* population that can overcome the resistance of all seven cowpea lines. Analysis of single sequence repeats of the *Striga* populations unraveled high differentiation and suggested that genetic relatedness is generally a result of geographic proximity rather than of host compatibility. This study indicates that generating a broad-spectrum and durable *S. gesnerioides* cowpea-resistant lines requires stacking of multiple resistance genes (Ohlson and Timko, 2020).

In a field study in Nigeria, Muranaka et al. (2011) evaluated the susceptibility of different cultivars toward *S*. *gesnerioides* race SG3, which confirmed the resistance of the cultivars B301, IT97K-499-35, and IT98K-205-8. In a further field study performed in Burkina Faso, Tignegre et al. (2013) identified 11 cowpea genotypes with resistance to several *S. gesnerioides* races. Similarly, Omoigui et al. (2017) identified two high-resistant varieties, that is, UAM09 1046-6-1 and UAM09 1046-6-2, in the dry savanna agro-system in Nigeria, by phenotypic screening and using biplot analysis.

### Cultural and agronomic practices

Trap cropping, sowing of false host such as cowpea, groundnut, sesame, and cotton to stimulate suicidal germination and to improve soil fertility were reported to be an effective way of seed bank depletion (Atera et al., 2013; Goldwasser and Rodenburg, 2013). Combining trap crops and nitrogen fertilizers was also reported to significantly decrease *Striga* seed bank (Tadesse, 2018). Cover cropping, sowing crops for the protection and enrichment of the soil showed *Striga* suppression directly through mulching, induction of suicidal germination, or its shading effect (Pickett et al., 2010; Goldwasser and Rodenburg, 2013; Randrianjafizanaka et al., 2018). Intercropping of cereals with legumes or a trap crop such as *Desmodium spp*. (Push–Pull) reduced *Striga* emergence by improving soil fertility, organic matter, and soil moisture content and releasing allelochemicals, such as C-glycosylflavonoids, isoflavanones, isoschaftoside, phenolics, 3,4-dihydroxybenzoic acid, which might impact *Striga* germination, growth, or development (Makoi and Ndakidemi, 2012; Khan et al., 2014; Pickett et al., 2014; Hooper et al., 2015; Midega et al., 2017; Hailu et al., 2018). A combination of herbicide-resistant maize varieties intercropped with legumes appeared more effective against *Striga* (Kanampiu et al., 2018).

Fallow and crop rotation were found not only to improve soil fertility and crop yield, but also to lower *Striga* infestation in Cameroon (Ayongwa et al., 2010). In a similar study, a reduction in *Striga* seed bank through fallowing was reported in Mali and Niger (Van Mourik et al., 2011). Rotation of a nonhost legume crop can considerably reduce the *Striga* seedbank in infested fields, leading to decreased infestation and significantly enhanced yield, compared with continuous cereal cultivation (Franke et al., 2018). Recently, Kountche et al. (2019) suggested field partitioning into two sections where suicidal germination agents (see below) and existing integrated *Striga* and soil management practices can be rotated to sustain *Striga* seed bank reduction. Hand pulling, uprooting *Striga* by hand or hand tools, is still considered as the cheapest traditional *Striga* control method (Goldwasser and Rodenburg, 2013). It is recommended to apply this method before *Striga* flowering, to prevent further seed setting and seed bank accumulation (Ayongwa et al., 2010; Sibhatu, 2016). However, hand weeding is laborious, time-consuming and less effective in reducing damage to standing crop (Mahuku et al., 2017). Fertilizer application and organic amendments showed a negative impact on *Striga* emergence (Ayongwa et al., 2011). The reduction of *Striga* infestation of rice upon fertilizer application is likely caused by a decrease in SL exudation (Jamil et al., 2011b). Similarly, nitrogen–phosphate–potassium fertilizer, micro-dosing of di-ammonium phosphate, and phosphate-based seed priming have been shown to reduce SL release and *Striga* parasitism in sorghum, pearl millet, and rice (Jamil et al., 2012b, 2014a, 2014b; Isah et al., 2013).

Recently, combining conservation agriculture practices, such as cover cropping and fertilizer applications, with *Striga*-resistant varieties was found to alleviate *Striga* impact on rice and maize (Abdallah et al., 2015; Rodenburg et al., 2020). Although most of the cultural practices are less expensive and helpful in reducing parasitic seed bank and improving soil fertility and soil texture, they are constrained by low farmer acceptance, the need for introducing additional crops, labor forces, and financial resources (Murage et al., 2011).

### Biocontrol by microbiome

Myco-herbicides developed from the fungus *Fusarium oxysporum* showed *Striga* inhibition by reducing its attachment to cereals and decreasing seed bank in infested soils (Rebeka et al., 2013; Zimmermann et al., 2016; Bàrberi, 2019). Some *F. oxysporum* strains produce high amounts of the amino acids l-leucine and l-tyrosin which are toxic to *Striga*—but not to maize—as they disrupt the tightly regulated free amino acid homeostasis. In addition, methionine released by *F. oxysporum* strains can be converted by soil microbes into the germination stimulant ethylene, causing suicidal germination of *Striga* seeds (Nzioki et al., 2016; Rubiales et al., 2018). Seed coating with *F. oxysporum* (FOXY2) was proposed as an effective way to deal with *Striga* under field conditions (Elzein et al., 2010; Ndambi et al., 2011, 2012; Rebeka et al., 2013; Watson, 2013). Later PSM197 and FOXY2 were encapsulated in a granular formulation (PESTA) for easy application/longevity, and a reduction of 75% in *Striga* emergence was observed in maize and sorghum crops (Schaub et al., 2006). FOXY2 was further classified as *F. oxysporum* f. *sp. strigae* (FOS) based on its highly selective inhibition of *S. asiatica* and *S. hermonthica* emergence (Mrema et al., 2018; 2020; Shayanowako et al., 2020). The characteristics that make mycoherbicides striking bioagents against *Striga* include host specificity, high aggressiveness, easy mass production, genetic diversity, and long storage life (Rebeka et al., 2013; Nzioki et al., 2016). Fungal delivery of primary inoculum on toothpick, multiplication of secondary field inoculum and farmers training are key components of this approach (Nzioki et al., 2016). The application of FOXY T14 led to an increase of 42%–56% in crop yield and reduction of 80% in *Striga* infestation under experimental conditions. However, *F. oxysporum* has not been extensively used in real fields, which might be due to a low effectiveness (Nzioki et al., 2016). Moreover, *F. oxysporum* might cause diseases, such as *Fusarium* wilt and dieback, in solanaceous crops (Zarafi et al., 2015).

Soil microbes like plant growth-promoting bacteria (PGPR), employment of AM fungi and some bacterial strains caused considerable reduction in *Striga* germination, attachment, and emergence (Lendzemo et al., 2009; Babalola, 2010; Hassan and Babiker, 2011; Mazaheri-Naeini et al., 2015). The potential of AM fungi in alleviating *Striga* infection has been indicated in a number of studies (Akiyama et al., 2005; Lendzemo et al., 2006; Bouwmeester et al., 2007; Xie et al., 2010). AM fungi not only enhance cereal growth and performance to withstand *Striga* damage but also facilitate host plant’s uptake of water, phosphorus (P), and micronutrients from the soil through the wide net of extraradical fungal hyphae (Bonfante and Genre, 2010). Increased uptake of P through symbiotic interaction by AM fungi could ultimately reduce SLs exudation by the host in the soil, thereby lowering *Striga* infection (Lendzemo et al., 2007; López-Ráez et al., 2011).

In a screening study, four PGPR suppressed *Striga* infestation in sorghum. Moreover, application of the strain *B. subtilis* GBO3 led to the death of 35%–59% of emerging *Striga* tubercles and to a 23% reduction in *Striga* attachment (Mounde et al., 2015). *Striga* seed germinating activity of sorghum root exudates decreased significantly upon treatment with *Pseudomonas* bacterial suspensions, which might be due to degradation of SLs (Ali et al., 2013). As shown for isolates of *Bacillus*, *Streptomyces*, and *Rhizobium* genera, the production of compounds with antibiotic activity and of extracellular enzymes, such as xylanases, pectinases, and amylases, can directly cause *Striga* seed decay (Neondo et al., 2017). In addition, soil microbes could impact *Striga* by releasing amino acids, such as tyrosine, leucine, and/or methionine (Vurro et al., 2009) or by producing secondary metabolites, such as anthranilic acid, β-lactone derivatives sesquiterpenoids, tricothecenes, which might interfere with SL perception (Tyc et al., 2017). Similarly, there is some evidence for SL degradation by fungi, which might lead to reduction in *Striga* germinating activity of released exudates (Boari et al., 2016). The germinating activity of root exudates and, hence, infestation by *Striga* can be also affected by soil microbes that modulate root architecture and growth (Huang et al., 2014).

The usage of soil microbes (AM fungi, PGPR, and other bacterial strains) is now considered as a promising, cost-effective, and environmentally safe approach for combating *Striga* (Watson, 2013; Samejima and Sugimoto, 2018). However, a number of biotic and abiotic factors can affect the efficacy of this approach, especially under field conditions. Finding a suitable inoculum medium, its mass production, suitable formulation, storage, shelf-life, consistency, and compatibility of applied microbes with the host, and the maintenance of their activity in infested soils must be taken into consideration. The validation and further development of this microbes-based biocontrol approach still requires intensive research and field testing under varying climatic and edaphic conditions (Müller‐Stöver et al., 2016; Mohammadi, 2019).

### Use of herbicides and suicidal agents

Seed coating of imazapyr-resistant maize with imidazolinone herbicides, such as imazapyr, imazapic, pyrithiobac, and imazaquin, caused a reduction in *Striga* emergence throughout the planting season and led to a three- to four-fold increase in maize yield (Menkir et al., 2010; Chikoye et al., 2011; Habimana et al., 2014; Makumbi et al., 2015). Similarly, treatment of cowpea seeds with imazaquin at 0.24 kg a.i. ha^−1^ significantly decreased *Striga* infection in different cowpea genotypes (Lado et al., 2018). Albeit promising results, availability of IR resistance seeds to the farmers, application technology, the risk of generating resistance in *Striga* itself, and the impact on environment are important issues that need to be considered (Ransom et al., 2012).

**Figure 2:**
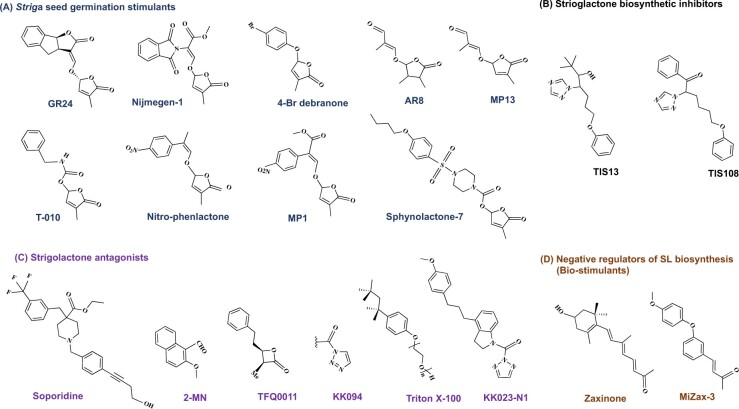
Structure of germination stimulants and further chemicals used in *Striga* control. A, Structure of the SL analogs GR24, Nijmegen-1, 4-Br debranone, AR8, MP13, Nitro-phenlactone, MP1, and Sphynolactone-7. B, Structure of TIS13, TIS108, two inhibitors of SL biosynthesis. C, Structure of the SL antagonists Soporidine, 2-methoxy-1-naphthaldehyde, TFQ0011, KK094, Triton X-100, and KK023-N1. D, Structure of zaxinone and its mimic MiZax3, which act as growth-promoting compounds (biostimulants) and negative regulator of SL biosynthesis at transcript level in rice.

Application of synthetic germination stimulants to deplete *Striga* seed bank in infested soils by inducing suicidal germination in the absence of host has recently gained a lot of attention (Samejima et al., 2016b; Zwanenburg et al., 2016; Kountche et al., 2019). Several SL analogs (Fig. 2) have been developed and evaluated (Xie et al., 2010; Mwakaboko and Zwanenburg, 2011; Kgosi et al., 2012; Boyer et al., 2014; Screpanti et al., 2016; Vurro et al., 2016; Jamil et al., 2018, 2020; Uraguchi et al., 2018; Prandi and McErlean, 2019). Functional tests revealed large variation in the efficiency of these analogs in exerting different SL functions, including the induction of *Striga* seed germination under lab conditions. Generally, SL analogs/mimics are quite variable in their structure but have in common the D-ring and ether bridge characteristic for natural SLs (Zwanenburg and Mwakaboko, 2011; Fukui et al., 2013, 2017; Cala et al., 2016; Takahashi et al., 2016; Oancea et al., 2017; Jamil et al., 2020). Structure–activity relationship studies have shown that minor structure modification can lead to significant changes in the biological activity (Cohen et al., 2013; Zwanenburg et al., 2013; Jamil et al., 2018, 2019, 2020). Following the discovery of the central SL biosynthesis intermediate carlactone (Alder et al., 2012), a carlactone-based SL analog, nitro-phenlactone was developed. Nitro-phenlactone showed high parasitic seed germinating activity in *Phelipanche ramosa*, which was at least comparable to GR24, but not in *Striga* (Jia et al., 2016). Subsequently, a series of carlactonoic acid-based analogs, called methyl-phenlactonoates (MPs), were developed and tested (Jamil et al., 2018, 2020). The three analogs MP1, MP3 and MP16 showed high suicidal germination activity on *S. hermonthica* in lab, greenhouse and field trials performed in Burkina Faso (Jamil et al., 2018, 2020; Kountche et al., 2019). Similarly, a carbamate-derived SL analog T-010 was applied in an infested field in Sudan, which led to a significant reduction in *Striga* emergence and better growth of sorghum (Samejima et al., 2016b). Recently, a chemical screen for SL agonists, followed by structural modifications, led to a highly active compound that triggers *Striga* seed germination under lab conditions at femtomolar range concentrations (Uraguchi et al., 2018). However, the question about the activity of this analog in field is still open.

Although a large number of synthetic stimulants have been developed and proposed for usage as suicidal germination agents, there are only very few studies that assess the practicability and success of the suicidal germination approach and the activity of these stimulants in infested fields (Samejima et al., 2016b; Zwanenburg et al., 2016; Kountche et al., 2019). Indeed, the evaluation of the synthetic-stimulant-based suicidal germination strategy requires an application protocol suitable for infested regions in Africa, in addition to efficient and easy-to-synthesize SL analogs. Such a protocol needs to include an appropriate formulation and to account for local conditions, including less developed infrastructure. Given the water scarcity in the Sahel zone, the realization of the suicidal germination concept in the arid and semi-arid *Striga*-infested regions in SSA requires also a method that does not consume large amounts of water. Recently, Kountche et al. (2019) established a pipeline for assessing the germinating activity of SL analogs, which includes tests under lab conditions and in greenhouse, and mini and large field trials in Africa. For application in field, they developed a protocol for rain-fed agriculture, which requires a minimum of water and exploits rainfall for diluting and distributing of sprayed SL analogs. Following this protocol, the application of three SL analogs, that is, MP1, MP3, Nijmegen-1, at a final concentration below one micromole led to an up to 55% and 65% reduction in *Striga* emergence in an infested sorghum and pearl millet field, respectively (Kountche et al., 2019). These results and the study of Samejima et al. (2016b) demonstrate that the suicidal germination is a practicable and promising strategy to deplete *Striga* seed banks in infested African fields.

### Development of novel chemicals for *Striga* control

Recent knowledge on SL biosynthesis and perception, and the availability of sequenced genomes have paved the way for developing new alternatives to combat root parasitic plants by chemicals, which decrease host SL release by inhibiting biosynthetic enzymes, down-regulating related transcripts, or blocking SL perception in the parasite.

Inhibitors of SL biosynthesis reported so far target either the formation of carotenoids, the precursor of SLs, or SL biosynthesis itself. Application of fluridone or norflurazon, which inhibit the desaturation of phytoene in carotenoid biosynthesis (Zheng et al., 2020), to rice plants decreased SL production and, hence, *Striga* germination (Jamil et al., 2010). However, treatment with carotenoid biosynthesis inhibitors may cause unwanted effects, given the diverse functions of carotenoids (Felemban et al., 2019; Zheng et al., 2020). Ito et al. (2011) developed the triazole-derivative TIS13 as inhibitor of SL biosynthesis and demonstrated its effect in decreasing rice SL release. On the basis of these results, a new set of structurally related SL biosynthesis inhibitor candidates were synthesized, including TIS108 that showed considerable reduction in the release of *Striga* germination stimulants without affecting the host (Ito et al., 2013). Further development of TIS108 led to KK05 that caused higher reduction of 4-deoxyorobanchol release in rice exudates (Kawada et al., 2019).

SL antagonists, which specifically inhibit *Striga* SL perception, are a promising tool that could be applied in the presence of the host, enabling *Striga* control throughout the cropping season and complementing the suicidal germination strategy employed in host’s absence. The development of specific germination inhibitors has become possible through the identification of *Striga* SL receptors, particularly the most sensitive one ShHTL7, which are involved in *Striga* seed germination and differ from the receptors mediating SL response in host plants (Toh et al., 2015). *Striga* SL receptors that regulate seed germination are supposed to have evolved from the homolog receptor KAI2 that perceives karrikins, smoke derived compounds mimicking a yet unidentified plant growth regulator inducing seed germination in nonparasitic plants (Toh et al., 2014; Waters et al., 2014; Conn and Nelson, 2016). Thus, the *Striga* ShHTL7 can replace KAI2 and mediate SL-dependent seed germination in Arabidopsis *kai2* mutant (Holbrook-Smith et al., 2016). This capability allowed the establishment of a high-throughput screening for chemicals that block the *Striga* ShHTL7 receptor and act as SL antagonists, which led to the identification of soporidine (SOP; Holbrook-Smith et al., 2016). Evaluation of SOP activity on *Striga* seed germination indicated its capability to block this process (Tsuchiya et al., 2018). A serendipity discovery of a potent SL antagonist that specifically blocks *Striga* ShHTL7 was recently reported (Hameed et al., 2018). The authors aimed at elucidating the structure of ShHTL7 by crystallography and found the receptor tightly bound to the detergent Triton X-100 usually used in protein purification. Functional studies confirmed that Triton X-100 is a SL antagonist that specifically blocks the *Striga* ShHTL7, but not host SL receptors, and reduce *Striga* infestation in greenhouse (Hameed et al., 2018). SOP and Triton X-100 can be considered as lead compounds for the development of efficient *Striga* herbicides that act by inhibiting seed germination. An improvement of Triton X-100 efficiency might be achieved by modifying it to become a covalently binding SL antagonist. Similarly, 2-methoxy-1-naphthaldehyde as an SL antagonist (Mashita et al., 2016) or simple β-lactones (TFQ0011) that act as covalent and high-efficient inhibitors of SL receptors have been developed based on the general SL perception mechanism (Xiang et al., 2017). Triazole urea compounds, such as KK094, that covalently bind to nonparasitic plant SL receptors have been recently produced and shown to efficiently inhibit rice SL perception (Nakamura et al., 2019). A combination of structural elements of Triton X-100 and KK094 or β-lactone inhibitors might lead to highly efficient *Striga*-specific herbicides. The recently developed Triton X-100/KK094 hybrid structure KK023-N1 (Figure 2) is an example for such compounds (Randa et al., manuscript under review).

Zaxinone is a natural growth-regulating apocarotenoid metabolite that has been recently shown to be required for normal rice growth and development. In addition, application of this compound promotes rice root growth and downregulates SL biosynthesis at the transcript level (Wang et al., 2019). A greenhouse study demonstrated that zaxinone application can alleviate *Striga* infestation (Wang et al., 2019). These activities point to zaxinone as a suitable candidate for reducing *Striga* infestation by accelerating host growth improving its performance and decreasing parasitic seed germination. Very recently, a series of easy-to-synthesize Mimics of Zaxinone (MiZax) have been developed and tested in lab and greenhouse. This study unraveled MiZax3 and MiZax5 as potent zaxinone mimics that promote rice growth, reduce SL release and decrease *Striga* infestation (Wang et al., 2020). The simple synthesis protocol and high efficacy make MiZax very promising candidates for field application.

### Integrated *Striga* management

Effective *Striga* management cannot be achieved by a single control method and requires the integration of different approaches (Figure 1; Ejeta, 2007; Sibhatu, 2016). For instance, complementing host resistance with the use of *F. oxysporum* caused effective *Striga* reduction (Mrema et al., 2020; Shayanowako et al., 2020). Similarly, the reduction in *Striga* infestation achieved through seed coating of imazapyr-resistant hybrid maize can be significantly further increased by exploiting maize *Striga*-resistance (Kamara et al., 2020). As a further example, a cereal-legume crop rotation can be combined with the application of synthetic germination stimulants to deplete accumulated seed bank in infested soils (Hailu et al., 2018; Kountche et al., 2019). It can be also anticipated that the integration of suicidal germination technology with *Striga*-specific herbicides and/or zaxinone analogs can be very effective in dealing with the *Striga* problem in African agriculture.

## Concluding remarks and perspectives

A number of *Striga* control strategies have been proposed and tested during the past decade; however, further research efforts are still needed to provide sustainable and effective solutions to the *Striga* problem (see Outstanding Questions).

First, further understanding of the molecular and genetic basis of host resistance and host–parasite interaction is needed to breed crops with durable resistance. Use of genomic resources and modern tools, such as targeted gene editing or mutation breeding, can translate this knowledge into resistant crops. Second, rotation and/or intercropping with false host are important and cost-effective components of *Striga* management. This approach can be a vital element in existing ISM in SSA. Third, farmers willingness, commitment and planning, labor, capital, and input availability in particular cropping system are important factors for the effectiveness of push–pull, seed coating, or toothpick/FOS technologies. Compatibility to climatic and soil factors, famers awareness, dissemination of information, and transfer of technology to smallholders’ farmers are essential factors that need further attention. Fourth, the effectiveness of novel chemicals (SL analogs, antagonists, bio-stimulants) might depend upon formulation and method and time of application. A suitable formulation of efficient compounds, mass scale, low-cost synthesis, and practical field application for rain-fed African agriculture are crucial, particularly for seed bank depletion by suicidal agents. Moreover, impact of these chemicals on soil fauna and flora, soil structure, persistency, and residual effects on environment must be investigated prior to their release for on-farm application. Last, but not least, a smart package of technology integrating *Striga*-resistant cultivars with either fertilizers, mycoherbicides, herbicide-based seed coating, or new chemicals still needs to be worked out to achieve complete and robust control of *Striga*.

## Notes

M.J. contributed to the conception and proposal of the article, interpreted the relevant literature, and designed the figures. B.A.K. took part in the revision, particularly in the host resistance and suicidal part of the article. S.A.B. substantially contributed in the conception, editing, and structuring of the article and revised it critically to make the final version.

The author responsible for distribution of materials integral to the findings presented in this article in accordance with the policy described in the Instructions for Author (https://academic.oup.com/plphys/pages/general-instructions) is: Salim Al-Babili (salim.babili@kaust.edu.sa).

## References

[kiab040-B1] Abdallah B , Saha H, Tsanuo M (2015) Control of *Striga asiatica* through the integration of legume cover crops and *Striga* resistant maize. Int J Pure Appl Sci Technol 29: 42–53

[kiab040-B2] Adewale SA , Badu-Apraku B, Akinwale RO, Paterne AA, Gedil M, Garcia-Oliveira AL (2020) Genome-wide association study of Striga resistance in early maturing white tropical maize inbred lines. BMC Plant Biol 20: 1–16 32393176 10.1186/s12870-020-02360-0 PMC7212567

[kiab040-B3] Akaogu IC , Badu-Apraku B, Adetimirin VO, Vroh-Bi I, Oyekunle M, Akinwale RO (2013) Genetic diversity assessment of extra-early maturing yellow maize inbreds and hybrid performance in *Striga*-infested and *Striga*-free environments. J Agric Sci 151: 519–537

[kiab040-B4] Akaogu IC , Badu-Apraku B, Tongoona P, Ceballos H, Gracen V, Offei SK, Dzidzienyo D (2019) Inheritance of *Striga hermonthica* adaptive traits in an early-maturing white maize inbred line containing resistance genes from *Zea diploperennis*. Plant Breed 138: 546–552

[kiab040-B5] Akiyama K , Matsuzaki K-i, Hayashi H (2005) Plant sesquiterpenes induce hyphal branching in arbuscular mycorrhizal fungi. Nature 435: 824–827 15944706 10.1038/nature03608

[kiab040-B6] Al-Babili S , Bouwmeester HJ (2015) Strigolactones, a novel carotenoid-derived plant hormone. Annu Rev Plant Biol 66: 161–186 25621512 10.1146/annurev-arplant-043014-114759

[kiab040-B7] Alder A , Jamil M, Marzorati M, Bruno M, Vermathen M, Bigler P, Ghisla S, Bouwmeester H, Beyer P, Al-Babili S (2012) The path from β-carotene to carlactone, a strigolactone-like plant hormone. Science 335: 1348–1351 22422982 10.1126/science.1218094

[kiab040-B8] Ali HA , Elamin HB, Dirar HA (2013) Biological control of *Striga hermonthica* Del. Benth: Screening for bacteria scavenging Strigol. Univ Africa J Sci 1: 106–119

[kiab040-B9] Amusan IO , Rich PJ, Menkir A, Housley T, Ejeta G (2008) Resistance to *Striga hermonthica* in a maize inbred line derived from *Zea diploperennis*. New Phytol 178: 157–166 18208472 10.1111/j.1469-8137.2007.02355.x

[kiab040-B10] Atera EA , Itoh K, Azuma T, Ishii T (2012a) Farmers' perspectives on the biotic constraint of *Striga hermonthica* and its control in western Kenya. Weed Biol Manag 12: 53–62

[kiab040-B11] Atera EA , Itoh K, Azuma T, Ishii T (2012b) Response of NERICA rice to *Striga hermonthica* infections in Western Kenya. Int J Agric Biol 14: 271–275

[kiab040-B12] Atera EA , Ishii T, Onyango JC, Itoh K, Azuma T (2013) *Striga* infestation in Kenya: Status, distribution and management options. Sustain Agric Res 2: 99–108

[kiab040-B13] Ayongwa G , Stomph T, Hoevers R, Ngoumou T, Kuyper T (2010) *Striga* infestation in northern Cameroon: Magnitude, dynamics and implications for management. NJAS-Wagen J Life Sci 57: 159–165

[kiab040-B14] Ayongwa GC , Stomph TJ, Kuyper TW (2011) Host-parasite dynamics of *Sorghum bicolor* and *Striga hermonthica*—the influence of soil organic matter amendments of different C:N ratio. Crop Prot 30: 1613–1622

[kiab040-B15] Babalola OO (2010) Beneficial bacteria of agricultural importance. Biotechnol Lett 32: 1559–1570 20635120 10.1007/s10529-010-0347-0

[kiab040-B16] Badu-Apraku B , Talabi AO, Fakorede MAB, Fasanmade Y, Gedil M, Magorokosho C, Asiedu R (2019) Yield gains and associated changes in an early yellow bi-parental maize population following genomic selection for *Striga* resistance and drought tolerance. BMC Plant Biol 19: 119–129 30953477 10.1186/s12870-019-1740-z PMC6451270

[kiab040-B17] Bàrberi P (2019) Ecological weed management in Sub-Saharan Africa: prospects and implications on other agroecosystem services. Adv Agron 156: 219–264

[kiab040-B18] Bebawi FF , Eplee RE, Harris CE, Norris RS (1984) Longevity of witchweed (*Striga asiatica*) seed. Weed Sci 32: 494–497

[kiab040-B19] Bellis ES , Kelly EA, Lorts CM, Gao HR, Deleo VL, Rouhan G,, Budden A, Bhaskara GB, Hu ZB, Muscarella R, et al. (2020) Genomics of sorghum local adaptation to a parasitic plant. Proc Natl Acad Sci USA 117: 4243–4251 32047036 10.1073/pnas.1908707117 PMC7049153

[kiab040-B20] Berner D , Kling J, Singh B (1995) *Striga* research and control. A perspective from Africa. Plant Dis 79: 652–660

[kiab040-B21] Boari A , Ciasca B, Pineda‐Martos R, Lattanzio VM, Yoneyama K, Vurro M (2016) Parasitic weed management by using strigolactone‐degrading fungi. Pest Manag Sci 72: 2043–2047 26757233 10.1002/ps.4226

[kiab040-B22] Bonfante P , Genre A (2010) Mechanisms underlying beneficial plant–fungus interactions in mycorrhizal symbiosis. Nat Commun 1: 1–11 20975705 10.1038/ncomms1046

[kiab040-B23] Botanga CJ , Timko MP (2005) Genetic structure and analysis of host and nonhost interactions of *Striga gesnerioides* (witchweed) from central Florida. Phytopathology 95: 1166–1173 18943469 10.1094/PHYTO-95-1166

[kiab040-B24] Botanga CJ , Timko MP (2007) Phenetic relationships among different races of Striga gesnerioides (Willd.) Vatke from West Africa. Genome 1365: 1351–1365 10.1139/g06-086 17426750

[kiab040-B25] Bouwmeester HJ , Roux C, Lopez-Raez JA, Becard G (2007) Rhizosphere communication of plants, parasitic plants and AM fungi. Trends Plant Sci 12: 224–230 17416544 10.1016/j.tplants.2007.03.009

[kiab040-B26] Boyer F-D , de Saint Germain A, Pouvreau J-B, Clavé G, Pillot J-P, Roux A, Rasmussen A, Depuydt S, Lauressergues D, dit Frey NF (2014) New strigolactone analogs as plant hormones with low activities in the rhizosphere. Mol Plant 7: 675–690 24249726 10.1093/mp/sst163

[kiab040-B27] Cala A , Ghooray K, Fernandez-Aparicio M, Molinillo JMG, Galindo JCG, Rubiales D, Macias FA (2016) Phthalimide-derived strigolactone mimics as germinating agents for seeds of parasitic weeds. Pest Manag Sci 72: 2069–2081 27218223 10.1002/ps.4323

[kiab040-B28] Cechin I , Press M (1993) Nitrogen relations of the sorghum‐*Striga hermonthica* host‐parasite association: germination, attachment and early growth. New Phytol 124: 681–687 33874434 10.1111/j.1469-8137.1993.tb03858.x

[kiab040-B29] Chikoye D , Fontem LA, Menkir A (2011) Seed coating herbicide tolerant maize hybrids with imazapyr for *Striga hermonthica* (Del.) Benth control in the West African savanna. J Food Agric Environ 9: 416–421

[kiab040-B30] Cissoko M , Boisnard A, Rodenburg J, Press MC, Scholes JD (2011) New Rice for Africa (NERICA) cultivars exhibit different levels of post-attachment resistance against the parasitic weeds *Striga hermonthica* and *Striga asiatica*. New Phytol 192: 952–963 21883232 10.1111/j.1469-8137.2011.03846.x

[kiab040-B31] Cohen M , Prandi C, Occhiato EG, Tabasso S, Wininger S, Resnick N, Steinberger Y, Koltai H, Kapulnik Y (2013) Structure–function relations of strigolactone analogs: activity as plant hormones and plant interactions. Mol Plant 6: 141–152 23220943 10.1093/mp/sss134

[kiab040-B32] Conn CE , Nelson DC (2016) Evidence that KARRIKIN-INSENSITIVE2 (KAI2) receptors may perceive an unknown signal that is not karrikin or strigolactone. Front Plant Sci 6: 1219 26779242 10.3389/fpls.2015.01219 PMC4705300

[kiab040-B33] Ejeta G (2007) Breeding for *Striga* resistance in sorghum: exploitation of an intricate host-parasite biology. Crop Sci 47: 216–227

[kiab040-B34] Elzein A , Heller A, Ndambi B, De Mol M, Kroschel J, Cadisch G (2010) Cytological investigations on colonization of sorghum roots by the mycoherbicide Fusarium oxysporum f. sp *striga*e and its implications for *Striga* control using a seed treatment delivery system. Biol Control 53: 249–257

[kiab040-B35] Emechebe A , Ellis-Jones J, Schulz S, Chikoye D, Douthwaite B, Kureh I, Tarawali G, Hussaini M, Kormawa P, Sanni A (2004) Farmers' perception of the *Striga* problem and its control in Northern Nigeria. Exp Agric 40: 215–232

[kiab040-B36] FAOSTAT (2018) Food and Agriculture Organization of the United Nations. http://www.fao.org/ (June, 2020)

[kiab040-B37] Felemban A , Braguy J, Zurbriggen MD, Al-Babili S (2019) Apocarotenoids involved in plant development and stress response. Front Plant Sci 10: 1–16. 31611895 10.3389/fpls.2019.01168 PMC6777418

[kiab040-B38] Franke A , Van den Brand G, Vanlauwe B, Giller K (2018) Sustainable intensification through rotations with grain legumes in Sub-Saharan Africa: a review. Agric Ecosyst Environ 261: 172–185 29970946 10.1016/j.agee.2017.09.029 PMC5946712

[kiab040-B39] Fukui K , Ito S, Asami T (2013) Selective mimics of strigolactone actions and their potential use for controlling damage caused by root parasitic weeds. Mol Plant 6: 88–99 23204501 10.1093/mp/sss138

[kiab040-B40] Fukui K , Yamagami D, Ito S, Asami T (2017) A taylor-made design of phenoxyfuranone-type strigolactone mimic. Front Plant Sci 8: 1–11 28676802 10.3389/fpls.2017.00936 PMC5477565

[kiab040-B41] Gasura E , Setimela P, Mabasa S, Rwafa R, Kageler S, Nyakurwa C (2019) Response of IITA maize inbred lines bred for *Striga hermonthica* resistance to *Striga asiatica* and associated resistance mechanisms in southern Africa. Euphytica 215: 1–15

[kiab040-B42] Gobena D , Shimels M, Rich PJ, Ruyter-Spira C, Bouwmeester H, Kanuganti S, Mengiste T, Ejeta G (2017) Mutation in sorghum LOW GERMINATION STIMULANT 1 alters strigolactones and causes *Striga* resistance. Proc Natl Acad Sci USA 114: 4471–4476 28396420 10.1073/pnas.1618965114 PMC5410831

[kiab040-B43] Goldwasser Y , Rodenburg J (2013) Integrated agronomic management of parasitic weed seed banks. *In* Joel DM, Gressel J, Musselman LJ, eds, Parasitic Orobanchaceae: Parasitic Mechanisms and Control Strategies. Springer New York, pp 393–413

[kiab040-B44] Gressel J , Hanafi A, Head G, Marasas W, Obilana AB, Ochanda J, Souissi T, Tzotzos G (2004) Major heretofore intractable biotic constraints to African food security that may be amenable to novel biotechnological solutions. Crop Prot 23: 661–689

[kiab040-B45] Goyet V , Wada S, Cui S, Wakatake T, Shirasu K, Montiel G, Simier P, Yoshida S (2019) Haustorium inducing factors for parasitic Orobanchaceae. Front Plant Sci 10: 1–8 31555315 10.3389/fpls.2019.01056 PMC6726735

[kiab040-B46] Gurney A , Slate J, Press M, Scholes J (2006) A novel form of resistance in rice to the angiosperm parasite *Striga hermonthica*. New Phytol 169: 199–208 16390431 10.1111/j.1469-8137.2005.01560.x

[kiab040-B47] Habimana S , Nduwumuremyi A, Chinama R (2014) Management of *Orobanche* in field crops: a review. J Soil Sci Plant Nutr 14: 43–62

[kiab040-B48] Hailu G , Niassy S, Zeyaur KR, Ochatum N, Subramanian S (2018) Maize-legume intercropping and Push-Pull for management of fall armyworm, stemborers, and *Striga* in Uganda. Agron J 110: 2513–2522

[kiab040-B49] Hameed US , Haider I, Jamil M, Kountche BA, Guo XR, Zarban RA, Kim D, Al-Babili S, Arold ST (2018) Structural basis for specific inhibition of the highly sensitive ShHTL7 receptor. EMBO Rep 19: 1–14 30021834 10.15252/embr.201745619 PMC6123649

[kiab040-B50] Hassan MM , Babiker AGT (2011) Effects of bacterial strains and isolates on in situ germination, subsequent developmental stage of *Striga hermonthica* onto sorghum roots. Adv Environ Biol **5**: 3263–3270

[kiab040-B51] Haussmann B , Hess D, Omanya G, Folkertsma R, Reddy B, Kayentao M, Welz H, Geiger H (2004) Genomic regions influencing resistance to the parasitic weed *Striga hermonthica* in two recombinant inbred populations of sorghum. Theor Appl Genet 109: 1005–1016 15160241 10.1007/s00122-004-1706-9

[kiab040-B52] Hearne SJ (2009) Control—the *Striga* conundrum. Pest Manag Sci 65: 603–614 19301299 10.1002/ps.1735

[kiab040-B53] Holbrook-Smith D , Toh S, Tsuchiya Y, McCourt P (2016) Small-molecule antagonists of germination of the parasitic plant *Striga hermonthica*. Nat Chem Biol 12: 724–729 27428512 10.1038/nchembio.2129

[kiab040-B54] Hooper AM , Caulfield JC, Hao B, Pickett JA, Midega CAO, Khan ZR (2015) Isolation and identification of *Desmodium* root exudates from drought tolerant species used as intercrops against *Striga hermonthica*. Phytochemistry 117: 380–387 26164239 10.1016/j.phytochem.2015.06.026 PMC4560159

[kiab040-B55] Huang K , Whitlock R, Press M, Scholes J (2012a) Variation for host range within and among populations of the parasitic plant *Striga hermonthica*. Heredity 108: 96–104 21731054 10.1038/hdy.2011.52 PMC3262869

[kiab040-B56] Huang K , Mellor KE, Paul SN, Lawson MJ, Mackey AJ, Timko MP (2012b) Global changes in gene expression during compatible and incompatible interactions of cowpea (*Vigna unguiculata* L.) with the root parasitic angiosperm *Striga gesnerioides*. BMC Genomics 13: 402–417 22900582 10.1186/1471-2164-13-402 PMC3505475

[kiab040-B57] Huang X-F , Chaparro JM, Reardon KF, Zhang R, Shen Q, Vivanco JM **(** 2014) Rhizosphere interactions: root exudates, microbes, and microbial communities. Botany 92: 267–275

[kiab040-B58] Isah K , Kumar N, Lagoke S, Atayese M (2013) Management of *Striga* hermonthica on sorghum (*Sorghum bicolor*) using arbuscular mycorrhizal fungi (*Glomus mosae*) and NPK fertilizer levels. Pak J Biol Sci 16: 1563–1568 24511701 10.3923/pjbs.2013.1563.1568

[kiab040-B59] Ito S , Umehara M, Hanada A, Kitahata N, Hayase H, Yamaguchi S, Asami T (2011) Effects of triazole derivatives on strigolactone levels and growth retardation in rice. PLos One 6: 1–5 10.1371/journal.pone.0021723 PMC3132747 21760901

[kiab040-B60] Ito S , Umehara M, Hanada A, Yamaguchi S, Asami T (2013) Effects of strigolactone-biosynthesis inhibitor TIS108 on Arabidopsis. Plant Signal Behav 8: 1–3 10.4161/psb.24193 PMC3907539 23511201

[kiab040-B61] Jamil M , Charnikhova T, Verstappen F, Bouwmeester H (2010) Carotenoid inhibitors reduce strigolactone production and *Striga hermonthica* infection in rice. Arch Biochem Biophys 504: 123–131 20732294 10.1016/j.abb.2010.08.005

[kiab040-B62] Jamil M , Rodenburg J, Charnikhova T, Bouwmeester HJ (2011a) Pre-attachment *Striga hermonthica* resistance of New Rice for Africa (NERICA) cultivars based on low strigolactone production. New Phytol 192: 964–975 21883233 10.1111/j.1469-8137.2011.03850.x

[kiab040-B63] Jamil M , Charnikhova T, Cardoso C, Jamil T, Ueno K, Verstappen F, Asami T, Bouwmeester HJ (2011b) Quantification of the relationship between strigolactones and *Striga hermonthica* infection in rice under varying levels of nitrogen and phosphorus. Weed Res 51: 373–385

[kiab040-B64] Jamil M , Charnikhova T, Houshyani B, van Ast A, Bouwmeester HJ (2012a) Genetic variation in strigolactone production and tillering in rice and its effect on *Striga hermonthica* infection. Planta 235: 473–484 21947621 10.1007/s00425-011-1520-y PMC3288373

[kiab040-B65] Jamil M , Kanampiu FK, Karaya H, Charnikhova T, Bouwmeester HJ (2012b) *Striga hermonthica* parasitism in maize in response to N and P fertilizers. Field Crops Res 134: 1–10

[kiab040-B66] Jamil M , Charnikhova T, Jamil T, Ali Z, Mohamed NEMA, Van Mourik T, Bouwmeester HJ (2014a) Influence of fertilizer microdosing on strigolactone production and *Striga hermonthica* parasitism in pearl millet. Int J Agric Biol 16: 935–940

[kiab040-B67] Jamil M , Charnikhova T, Verstappen F, Ali Z, Wainwright H, Bouwmeester HJ (2014b) Effect of phosphate-based seed priming on strigolactone production and *Striga hermonthica* infection in cereals. Weed Res 54: 307–313

[kiab040-B68] Jamil M , Kountche BA, Haider I, Guo XJ, Ntui VO, Jia KP, Ali S, Hameed US, Nakamura H, Lyu Y, et al. (2018) Methyl phenlactonoates are efficient strigolactone analogs with simple structure. J Exp Bot 69: 2319–2331 29300919 10.1093/jxb/erx438 PMC5913645

[kiab040-B69] Jamil M , Kountche BA, Haider I, Wang JY, Aldossary F, Zarban RA, Jia KP, Yonli D, Hameed UFS, Takahashi I, et al. (2019) Methylation at the C-3' in D-Ring of strigolactone analogs reduces biological activity in root parasitic plants and rice. Front Plant Sci 10: 1–14 31001294 10.3389/fpls.2019.00353 PMC6455008

[kiab040-B70] Jamil M , Kountche BA, Wang JY, Haider I, Jia K-P, Takahashi I, Ota T, Asami T, Al-Babili S (2020) A new series of carlactonoic acid based strigolactone analogs for fundamental and applied research. Front Plant Sci 11: 1–13 32373143 10.3389/fpls.2020.00434 PMC7179673

[kiab040-B71] Jia K-P , Kountche BA, Jamil M, Guo X, Ntui VO, Rüfenacht A, Rochange S, Al-Babili S (2016) Nitro-phenlactone, a carlactone analog with pleiotropic strigolactone activities. Mol Plant 9: 1341–1344 27288318 10.1016/j.molp.2016.05.017

[kiab040-B72] Joel DM , Bar H (2013) The seed and the seedling. *In* Joel DM, Gressel J, Musselman LJ, eds, Parasitic Orobanchaceae: Parasitic Mechanisms and Control Strategies. Springer New York, pp 147–165

[kiab040-B73] Joel DM (2000) The long-term approach to parasitic weeds control: manipulation of specific developmental mechanisms of the parasite. Crop Prot 19: 753–758

[kiab040-B74] Kamara AY , Menkir A, Chikoye D, Solomon R, Tofa AI, Omoigui LO (2020) Seed dressing maize with imazapyr to control *Striga hermonthica* in farmers' fields in the savannas of Nigeria. Agriculture Basel 10: 1–9

[kiab040-B75] Kanampiu F , Makumbi D, Mageto E, Omanya G, Waruingi S, Musyoka P, Ransom J (2018) Assessment of management options on *Striga* infestation and maize grain yield in Kenya. Weed Sci 66: 516–524 33583963 10.1017/wsc.2018.4 PMC7797635

[kiab040-B76] Karaya H , Njoroge K, Mugo S, Ariga ES, Kanampiu F, Nderitu JH (2012) Determination of levels of *Striga* germination stimulants for maize gene bank accessions and elite inbred lines. Int J Plant Prod 6: 209–223

[kiab040-B77] Kawada K , Takahashi I, Arai M, Sasaki Y, Asami T, Yajima S, Ito S (2019) Synthesis and biological evaluation of novel triazole derivatives as strigolactone biosynthesis inhibitors. J Agric Food Chem 67: 6143–6149 31083983 10.1021/acs.jafc.9b01276

[kiab040-B78] Kgosi RL , Zwanenburg B, Mwakaboko AS, Murdoch AJ (2012) Strigolactone analogues induce suicidal seed germination of *Striga* spp. in soil. Weed Res 52: 197–203

[kiab040-B79] Khan ZR , Midega CAO, Pittchar JO, Murage AW, Birkett MA, Bruce TJA, Pickett JA (2014) Achieving food security for one million sub-Saharan African poor through push-pull innovation by 2020. Philos Trans R Soc B Biol Sci 369: 1–11 10.1098/rstb.2012.0284 PMC3928888 24535391

[kiab040-B80] Kountche BA , Hash CT, Dodo H, Laoualy O, Sanogo MD, Timbeli A, Vigouroux Y, This D, Nijkamp R, Haussmann BIG (2013) Development of a pearl millet *Striga*-resistant genepool: Response to five cycles of recurrent selection under *Striga*-infested field conditions in West Africa. Field Crops Res 154: 82–90

[kiab040-B81] Kountche BA , Jamil M, Yonli D, Nikiema MP, Blanco‐Ania D, Asami T, Zwanenburg B, Al‐Babili S (2019) Suicidal germination as a control strategy for *Striga hermonthica* (Benth.) in smallholder farms of sub‐Saharan Africa. Plants People Planet 1: 107–118

[kiab040-B82] Lado A , Hussaini MA, Kamara AY (2018) Effectiveness of imazaquin seed treatment on *Striga* gesnerioides control and growth traits of seven cowpea genotypes. J Plant Pathol 100: 477–484

[kiab040-B83] Lendzemo VW , van Ast A, Kuyper TW (2006) Can arbuscular mycorrhizal fungi contribute to *Striga management* on cereals in Africa? Outlook Agric 35: 307–311

[kiab040-B84] Lendzemo VW , Kuyper TW, Matusova R, Bouwmeester HJ, Ast Av (2007) Colonization by arbuscular mycorrhizal fungi of sorghum leads to reduced germination and subsequent attachment and emergence of *Striga hermonthica*. Plant Signal Behav 2: 58–62 19516969 10.4161/psb.2.1.3884 PMC2633899

[kiab040-B85] Lendzemo V , Kuyper T, Vierheilig H (2009) *Striga* seed-germination activity of root exudates and compounds present in stems of Striga host and nonhost (trap crop) plants is reduced due to root colonization by arbuscular mycorrhizal fungi. Mycorrhiza 19: 287–294 19238457 10.1007/s00572-009-0235-4

[kiab040-B86] Li J , Timko MP (2009) Gene-for-gene resistance in Striga-cowpea associations. Science 325: 1094–1094 19713520 10.1126/science.1174754

[kiab040-B87] López-Ráez JA , Charnikhova T, Fernández I, Bouwmeester H, Pozo MJ (2011) Arbuscular mycorrhizal symbiosis decreases strigolactone production in tomato. J Plant Physiol 168: 294–297 20934776 10.1016/j.jplph.2010.08.011

[kiab040-B88] Mahuku G , Wosula E, Kanampiu F (2017) Integrated Pest Management in tropical cereal crops. In Rapisarda C, Cocuzza GEM, eds, Integrated Pest Management in Tropical Regions. CAB International, UK, pp 47–74

[kiab040-B89] Makoi JH , Ndakidemi PA (2012) Allelopathy as protectant, defence and growth stimulants in legume cereal mixed culture systems. N Z J Crop Hortic Sci 40: 161–186

[kiab040-B90] Makumbi D , Diallo A, Kanampiu F, Mugo S, Karaya H (2015) Agronomic performance and genotype×environment interaction of herbicide‐resistant maize varieties in eastern Africa. Crop Sci 55: 540–555

[kiab040-B91] Mandumbu R , Mutengwa C, Mabasa S, Mwenje E (2019) Challenges to the exploitation of host plant resistance for *Striga* management in cereals and legumes by farmers in sub-Saharan Africa: a review. Acta Agric Scand B Soil Plant Sci 69: 82–88

[kiab040-B92] Mashita O , Koishihara H, Fukui K, Nakamura H, Asami T (2016) Discovery and identification of 2-methoxy-1-naphthaldehyde as a novel strigolactone-signaling inhibitor. J Pestic Sci 41: 71–78 30363101 10.1584/jpestics.D16-028 PMC6140645

[kiab040-B93] Mazaheri-Naeini M , Sabbagh SK, Martinez Y, Sejalon-Delmas N, Roux C (2015) Assessment of Ustilago maydis as a fungal model for root infection studies. Fungal Biol 119: 145–153 25749366 10.1016/j.funbio.2014.12.002

[kiab040-B94] Mbuvi DA , Masiga CW, Kuria E, Masanga J, Wamalwa M, Mohamed A, Odeny D, Hamza N, Timko MP, Runo S (2017) Novel sources of witchweed (*Striga*) resistance from wild sorghum accessions. Front Plant Sci 8: 1–15 28220136 10.3389/fpls.2017.00116 PMC5292437

[kiab040-B95] Menkir A , Chikoye D, Lum F (2010) Incorporating an herbicide resistance gene into tropical maize with inherent polygenic resistance to control Striga hermonthica (Del.) Benth. Plant Breed 129: 385–392

[kiab040-B96] Menkir A , Meseka S (2019) Genetic improvement in resistance to *Striga* in tropical maize hybrids. Crop Sci 59: 2484–2497

[kiab040-B97] Midega CAO , Pickett J, Hooper A, Pittchar J, Khan ZR (2016) Maize landraces are less affected by *Striga hermonthica* relative to hybrids in Western Kenya. Weed Technol 30: 21–28

[kiab040-B98] Midega CA , Wasonga CJ, Hooper AM, Pickett JA, Khan ZR (2017) Drought-tolerant *Desmodium* species effectively suppress parasitic *Striga* weed and improve cereal grain yields in western Kenya. Crop Prot 98: 94–101 28775391 10.1016/j.cropro.2017.03.018 PMC5465939

[kiab040-B99] Mohamed KI , Papes M, Williams R, Benz BW, Peterson AT (2006) Global invasive potential of 10 parasitic witchweeds and related Orobanchaceae. AMBIO 35: 281–288 17240760 10.1579/05-r-051r.1

[kiab040-B100] Mohamed AH , Housley TL, Ejeta G (2010a) Inheritance of hyper sensitive response to *Striga* parasitism in sorghum *Sorghum bicolor* (L.) Moench. Afr J Agric Res 5: 2720–2729

[kiab040-B101] Mohamed AH , Housley TL, Ejeta G (2010b) An in vitro technique for studying specific *Striga* resistance mechanisms in sorghum. Afr J Agric Res 5: 1868–1875

[kiab040-B102] Mohammadi G (2019) Can soil microorganisms reduce Broomrape (Orobanche spp.) infestation in cropping systems?Microbiome in Plant Health and Disease. Springer, pp 385–402

[kiab040-B103] Mohemed N , Charnikhova T, Fradin EF, Rienstra J, Babiker AGT, Bouwmeester HJ (2018) Genetic variation in Sorghum bicolor strigolactones and their role in resistance against *Striga hermonthica*. J Exp Bot 69: 2415–2430 29415281 10.1093/jxb/ery041 PMC6498397

[kiab040-B104] Moumouni K , Kountche B, Jean M, Hash C, Vigouroux Y, Haussmann B, Belzile F (2015) Construction of a genetic map for pearl millet, *Pennisetum glaucum* (L.) R. Br., using a genotyping-by-sequencing (GBS) approach. Mol Breed 35: 1–5

[kiab040-B105] Mounde LG , Boh MY, Cotter M, Rasche F (2015) Potential of rhizobacteria for promoting sorghum growth and suppressing *Striga hermonthica* development. J Plant Dis Prot 122: 100–106

[kiab040-B106] Mrema E , Shimelis H, Laing M, Mwadzingeni L (2018) Genetic analysis of the maximum germination distance of *Striga* under *Fusarium oxysporum f. sp strigae* biocontrol in sorghum. J Integr Agric 17: 1585–1593

[kiab040-B107] Mrema E , Shimelis H, Laing M (2020) Combining ability of yield and yield components among *Fusarium oxysporum f. sp. Strigae*-compatible and *Striga*-resistant sorghum genotypes. Acta Agric Scand B Soil Plant Sci 70: 95–108

[kiab040-B108] Müller‐Stöver D , Nybroe O, Baraibar B, Loddo D, Eizenberg H, French K, Sønderskov M, Neve P, Peltzer D, Maczey N (2016) Contribution of the seed microbiome to weed management. Weed Res 56: 335–339

[kiab040-B109] Murage AW , Obare G, Chianu J, Amudavi DM, Pickett J, Khan ZR (2011) Duration analysis of technology adoption effects of dissemination pathways: a case of ‘push-pull’ technology for control of *Striga* weeds and stemborers in Western Kenya. Crop Prot 30: 531–538

[kiab040-B110] Muranaka S , Fatokun C, Boukar O (2011) Stability of *Striga gesnerioides* resistance mechanism in cowpea under high-infestation level, low soil fertility and drought stresses. J Food Agric Environ 9: 313–318

[kiab040-B111] Mutinda SM , Masanga J,, Mutuku JM, Runo S, Alakonya A (2018) KSTP 94, an open-pollinated maize variety has postattachment resistance to purple witchweed (*Striga hermonthica*). Weed Sci 66: 525–529

[kiab040-B112] Mutuku JM , Yoshida S, Shimizu T, Ichihashi Y, Wakatake T, Takahashi A, Seo M, Shirasu K (2015) The WRKY45-dependent signaling pathway is required for resistance against *Striga hermonthica* parasitism. Plant Physiol 168: 1152–1163 26025049 10.1104/pp.114.256404 PMC4741350

[kiab040-B113] Mutuku JM , Cui S, Hori C, Takeda Y, Tobimatsu Y, Nakabayashi R, Mori T, Saito K, Demura T, Umezawa T (2019) The structural integrity of lignin is crucial for resistance against *Striga hermonthica* parasitism in rice. Plant Physiol 179: 1796–1809 30670602 10.1104/pp.18.01133 PMC6446757

[kiab040-B114] Mwakaboko AS , Zwanenburg B (2011) Single step synthesis of strigolactone analogues from cyclic keto enols, germination stimulants for seeds of parasitic weeds. Bioorg Med Chem 19: 5006–5011 21757362 10.1016/j.bmc.2011.06.057

[kiab040-B115] Nakamura H , Hirabayashi K, Miyakawa T, Kikuzato K, Hu W, Xu Y, Jiang K, Takahashi I, Niiyama R, Dohmae N (2019) Triazole ureas covalently bind to strigolactone receptor and antagonize strigolactone responses. Mol Plant 12: 44–58 30391752 10.1016/j.molp.2018.10.006

[kiab040-B116] Ndambi B , Cadisch G, Elzein A, Heller A (2011) Colonization and control of *Striga hermonthica* by *Fusarium oxysporum f. sp strigae*, a mycoherbicide component: an anatomical study. Biol Control 58: 149–159

[kiab040-B117] Ndambi B , Cadisch G, Elzein A, Heller A (2012) Tissue specific reactions of sorghum roots to the mycoherbicide *Fusarium oxysporum f. sp strigae* versus the pathogenic *F. proliferatum*. Biocontrol Sci Technol 22: 135–150

[kiab040-B118] Neondo JO , Alakonya AE, Kasili RW (2017) Screening for potential *Striga hermonthica* fungal and bacterial biocontrol agents from suppressive soils in Western Kenya. BioControl 62: 705–717

[kiab040-B119] Nickrent DL , Musselman LJ (2004) Introduction to parasitic flowering plants. Plant Health Instr 13: 300–315

[kiab040-B120] Nyakurwa CS , Gasura E, Setimela PS, Mabasa S, Rugare JT, Mutsvanga S (2018) Reaction of new quality protein maize genotypes to *Striga asiatica*. Crop Sci 58: 1201–1218

[kiab040-B121] Nzioki HS , Oyosi F, Morris CE, Kaya E, Pilgeram AL, Baker CS, Sands DC (2016) *Striga* biocontrol on a Toothpick: a readily deployable and inexpensive method for smallholder farmers. Front Plant Sci 7: 1–8 27551284 10.3389/fpls.2016.01121 PMC4976096

[kiab040-B122] Oancea F , Georgescu E, Matusova R, Georgescu F, Nicolescu A, Raut I, Jecu M-L, Vladulescu M-C, Vladulescu L, Deleanu C (2017) New strigolactone mimics as exogenous signals for rhizosphere organisms. Molecules 22: 1–15 10.3390/molecules22060961 PMC6152683 28598371

[kiab040-B123] Ohlson EW , Timko MP (2020) Race structure of cowpea witchweed (*Striga gesnerioides*) in West Africa and its implications for *Striga* resistance breeding of cowpea. Weed Sci 68: 125–133

[kiab040-B124] Omoigui LO , Kamara AY, Ajeigbe HA, Akinwale RO, Timko MP, Oyekunle M, Bello LL (2017) Performance of cowpea varieties under *Striga gesnerioides* (Willd.) Vatke infestation using biplot analysis. Euphytica 213: 1–16

[kiab040-B125] Parker C (2009) Observations on the current status of *Orobanche* and *Striga* problems worldwide. Pest Manag Sci 65: 453–459 19206075 10.1002/ps.1713

[kiab040-B126] Parker C (2012) Parasitic weeds: a world challenge. Weed Sci 60: 269–276

[kiab040-B127] Pennisi E (2010) Armed and dangerous. Science 327: 804–805 20150482 10.1126/science.327.5967.804

[kiab040-B128] Pickett JA , Hamilton ML, Hooper AM, Khan ZR, Midega CA (2010) Companion cropping to manage parasitic plants. Annu Rev Phytopathol 48: 161–177 20429664 10.1146/annurev-phyto-073009-114433

[kiab040-B129] Pickett JA , Woodcock CM, Midega CA, Khan ZR (2014) Push–pull farming systems. Curr Opin Biotechnol 26: 125–132 24445079 10.1016/j.copbio.2013.12.006

[kiab040-B130] Prandi C , McErlean CSP (2019) The chemistry of strigolactones. In Koltai H, Prandi C, eds, Strigolactones—Biology and Applications, Vol 1. Springer International Publishing, Springer Nature Switzerland AG, pp 163–198

[kiab040-B131] Randrianjafizanaka MT , Autfray P, Andrianaivo AP, Ramonta IR, Rodenburg J (2018) Combined effects of cover crops, mulch, zero-tillage and resistant varieties on *Striga asiatica* (L.) Kuntze in rice-maize rotation systems. Agric Ecosyst Environ 256: 23–33

[kiab040-B132] Ransom J , Kanampiu F, Gressel J, De Groote H, Burnet M, Odhiambo G (2012) Herbicide applied to imidazolinone resistant-maize seed as a *Striga* control option for small-scale African farmers. Weed Sci 60: 283–289

[kiab040-B133] Rebeka G , Shimelis H, Laing MD, Tongoona P, Mandefro N (2013) Evaluation of sorghum genotypes compatibility with *Fusarium oxysporum* under *Striga* infestation. Crop Sci 53: 385–393

[kiab040-B134] Rodenburg J , Riches CR, Kayeke JM (2010) Addressing current and future problems of parasitic weeds in rice. Crop Prot 29: 210–221

[kiab040-B135] Rodenburg J , Cissoko M, Kayeke J,, Dieng I, Khan ZR, Midega CAO, Onyuka EA, Scholes JD (2015) Do NERICA rice cultivars express resistance to *Striga hermonthica* (Del.) Benth and *Striga asiatica* (L.) Kuntze under field conditions? Field Crops Res 170: 83–94 26089591 10.1016/j.fcr.2014.10.010 PMC4459690

[kiab040-B136] Rodenburg J , Demont M, Zwart SJ, Bastiaans L (2016) Parasitic weed incidence and related economic losses in rice in Africa. Agric Ecosyst Environ 235: 306–317

[kiab040-B137] Rodenburg J , Cissoko M, Kayongo N, Dieng I, Bisikwa J, Irakiza R, Masoka I, Midega CAO, Scholes JD (2017) Genetic variation and host-parasite specificity of *Striga* resistance and tolerance in rice: The need for predictive breeding. New Phytol 214: 1267–1280 28191641 10.1111/nph.14451 PMC5412873

[kiab040-B138] Rodenburg J , Randrianjafizanaka MT, Büchi L, Dieng I, Andrianaivo AP, Ravaomanarivo LHR, Autfray P (2020) Mixed outcomes from conservation practices on soils and *Striga*-affected yields of a low-input, rice–maize system in Madagascar. Agron Sustain Dev 40: 1–11

[kiab040-B139] Rubiales D , Fernández-Aparicio M, Vurro M, Eizenberg H (2018) Advances in parasitic weed research. Front Plant Sci 9: 236–239 29563922 10.3389/fpls.2018.00236 PMC5846014

[kiab040-B140] Samejima H , Babiker AG, Mustafa A, Sugimoto Y (2016a) Identification of Striga hermonthica-resistant upland rice varieties in Sudan and their resistance phenotypes. Front Plant Sci 7: 634–646 27242837 10.3389/fpls.2016.00634 PMC4865650

[kiab040-B141] Samejima H , Babiker AG, Takikawa H, Sasaki M, Sugimoto Y (2016b) Practicality of the suicidal germination approach for controlling *Striga hermonthica*. Pest Manag Sci 72: 2035–2042 26732430 10.1002/ps.4215

[kiab040-B142] Samejima H , Sugimoto Y (2018) Recent research progress in combatting root parasitic weeds. Biotechnol Biotechnol Equip 32: 221–240

[kiab040-B143] Satish K , Gutema Z, Grenier C, Rich PJ, Ejeta G (2012) Molecular tagging and validation of microsatellite markers linked to the low germination stimulant gene (lgs) for Striga resistance in sorghum Sorghum bicolor (L.) Moench. Theor Appl Genet 124: 989–1003 22159758 10.1007/s00122-011-1763-9

[kiab040-B144] Sattler FT , Sanogo MD, Kassari IA, Angarawai II, Gwadi KW, Dodo H, Haussmann BIG (2018) Characterization of West and Central African accessions from a pearl millet reference collection for agro-morphological traits and Striga resistance. Plant Genet Resour 16: 260–272

[kiab040-B145] Schaub B , Marley P, Elzein A, Kroschel J (2006) Field evaluation of an integrated *Striga hermonthica* management in Sub-Saharan Africa: synergy between *Striga*-mycoherbicides (biocontrol) and sorghum and maize resistant varieties. J Plant Dis Prot 20: 691–699

[kiab040-B146] Scholes JD , Press MC (2008) *Striga* infestation of cereal crops—an unsolved problem in resource limited agriculture. Curr Opin Plant Biol 11: 180–186 18337158 10.1016/j.pbi.2008.02.004

[kiab040-B147] Screpanti C , Fonné-Pfister R, Lumbroso A, Rendine S, Lachia M, De Mesmaeker A (2016) Strigolactone derivatives for potential crop enhancement applications. Bioorg Med Chem Lett 26: 2392–2400 27036522 10.1016/j.bmcl.2016.03.072

[kiab040-B148] Shayanowako AI , Shimelis H, Laing MD, Mwadzingeni L (2020) *Striga* resistance and compatibility of maize genotypes to a biocontrol agent, *Fusarium oxysporum f. sp. strigae*. J Crop Improv 1: 1–18

[kiab040-B149] Shen H , Ye W, Hong L, Huang H, Wang Z, Deng X, Yang Q, Xu Z (2006) Progress in parasitic plant biology: host selection and nutrient transfer. Plant Biol 8: 175–185 16547862 10.1055/s-2006-923796

[kiab040-B150] Sibhatu B (2016) Review on *Striga* weed management. Int J Life Sci Scienti Res 2: 110–120

[kiab040-B151] Su C , Liu H, Wafula EK, Honaas L, de Pamphilis CW, Timko MP (2020) SHR4z, a novel decoy effector from the haustorium of the parasitic weed *Striga gesnerioides*, suppresses host plant immunity. New Phytol 226: 891–908 31788811 10.1111/nph.16351 PMC7187149

[kiab040-B152] Swarbrick P , Huang K, Liu G, Slate J, Press M, Scholes J (2008) Global patterns of gene expression in rice cultivars undergoing a susceptible or resistant interaction with the parasitic plant *Striga hermonthica*. New Phytol 179: 515–529 19086183 10.1111/j.1469-8137.2008.02484.x

[kiab040-B153] Swarbrick PJ , Scholes JD, Press MC, Slate J (2009) A major QTL for resistance of rice to the parasitic plant *Striga hermonthica* is not dependent on genetic background. Pest Manag Sci 65: 528–532 19222023 10.1002/ps.1719

[kiab040-B154] Tadesse F (2018) Effect of Striga trap crops and nitrogen fertilizer application on yield and yield related traits of Sorghum [Sorghum bicolor (L.) Moench] at Fedis District, Eastern Ethiopia. Open Access Lib J 5: 1–17

[kiab040-B155] Takahashi I , Fukui K, Asami T (2016) Chemical modification of a phenoxyfuranone-type strigolactone mimic for selective effects on rice tillering or *Striga hermonthica* seed germination. Pest Manag Sci 72: 2048–2053 26929041 10.1002/ps.4265

[kiab040-B156] Tank DC , Beardsley PM, Kelchner SA, Olmstead RG **(** 2006) Review of the systematics of Scrophulariaceae s.l. and their current disposition. Aust Syst Bot 19: 289–307

[kiab040-B157] Tignegre JBS , Ouedraogo JT, Melis R, Tongoona P, Sibiya J, Makanda I, Drabo I (2013) Identification of new sources of resistance to *Striga gesnerioides* in cowpea germplasm. Plant Breed 132: 330–336

[kiab040-B158] Timko MP , Singh B (2008) Cowpea, a multifunctional legume. Genomics of Tropical Crop Plants. Springer, New York, USA, pp 227–258

[kiab040-B159] Timko MP , Huang K, Lis KE (2012) Host resistance and parasite virulence in *Striga*–host plant interactions: a shifting balance of power. Weed Sci 60: 307–315

[kiab040-B160] Toh S , Holbrook-Smith D, Stokes ME, Tsuchiya Y, McCourt P (2014) Detection of parasitic plant suicide germination compounds using a high-throughput Arabidopsis HTL/KAI2 strigolactone perception system. Chem Biol 21: 988–998 25126711 10.1016/j.chembiol.2014.07.005

[kiab040-B161] Toh S , Holbrook-Smith D, Stogios PJ, Onopriyenko O, Lumba S, Tsuchiya Y, Savchenko A, McCourt P (2015) Structure-function analysis identifies highly sensitive strigolactone receptors in *Striga*. Science 350: 203–207 26450211 10.1126/science.aac9476

[kiab040-B162] Tsuchiya Y , Yoshimura M, Hagihara S (2018) The dynamics of strigolactone perception in *Striga hermonthica*: a working hypothesis. J Exp Bot 69: 2281–2290 29474634 10.1093/jxb/ery061

[kiab040-B163] Tyc O , Song C, Dickschat JS, Vos M, Garbeva P (2017) The ecological role of volatile and soluble secondary metabolites produced by soil bacteria. Trends Microbiol 25: 280–292 28038926 10.1016/j.tim.2016.12.002

[kiab040-B164] Uraguchi D , Kuwata K, Hijikata Y, Yamaguchi R, Imaizumi H, Sathiyanarayanan AM, Rakers C, Mori N, Akiyama K, Irle S, et al. (2018) A femtomolar-range suicide germination stimulant for the parasitic plant *Striga hermonthica*. Science 362: 1301–1305 30545887 10.1126/science.aau5445

[kiab040-B165] Van Mourik TA , Stomph TJ, Murdoch AJ (2011) Purple witchweed (*Striga hermonthica*) germination and seedbank depletion under different crops, fallow, and bare soil. Weed Biol Manag 11: 100–110

[kiab040-B166] Vurro M , Boari A, Evidente A, Andolfi A, Zermane N (2009) Natural metabolites for parasitic weed management. Pest Manag Sci 65: 566–571 19266492 10.1002/ps.1742

[kiab040-B167] Vurro M , Prandi C, Baroccio F (2016) Strigolactones: how far is their commercial use for agricultural purposes? Pest Manag Sci 72: 2026–2034 26869010 10.1002/ps.4254

[kiab040-B168] Wakabayashi T , Hamana M, Mori A, Akiyama R, Ueno K, Osakabe K, Osakabe Y, Suzuki H, Takikawa H, Mizutani M (2019) Direct conversion of carlactonoic acid to orobanchol by cytochrome P450 CYP722C in strigolactone biosynthesis. Sci Adv 5: 1–10 10.1126/sciadv.aax9067 PMC6989309 32064317

[kiab040-B169] Wakabayashi T , Shida K, Kitano Y, Takikawa H, Mizutani M, Sugimoto Y (2020) CYP722C from Gossypium arboreum catalyzes the conversion of carlactonoic acid to 5-deoxystrigol. Planta 251: 1–6 10.1007/s00425-020-03390-6 32306106

[kiab040-B170] Wang JY , Haider I, Jamil M, Fiorilli V, Saito Y, Mi J, Baz L, Kountche BA, Jia K-P, Guo X (2019) The apocarotenoid metabolite zaxinone regulates growth and strigolactone biosynthesis in rice. Nat Commun 10: 1–9 30778050 10.1038/s41467-019-08461-1 PMC6379432

[kiab040-B171] Wang JY , Jamil M, Lin P-Y, Ota T, Fiorilli V, Novero M, Zarban RA, Kountche BA, Takahashi I, Martínez C (2020) Efficient mimics for elucidating zaxinone biology and promoting agricultural applications. Mol Plant 13: 1654–1661 32835886 10.1016/j.molp.2020.08.009 PMC7656291

[kiab040-B172] Waters MT , Scaffidi A, Sun YK, Flematti GR, Smith SM (2014) The karrikin response system of Arabidopsis. Plant J 79: 623–631 24433542 10.1111/tpj.12430

[kiab040-B173] Watson AK (2013) Biocontrol. In Joel Daniel M., Gressel Jonathan, Musselman LJ, eds, Parasitic Orobanchaceae: Parasitic Mechanisms and Control Strategies, Vol 1. Springer, New York, pp 469–497

[kiab040-B174] Wilson J , Hess D, Hanna W (2000) Resistance to *Striga hermonthica* in wild accessions of the primary gene pool of Pennisetum glaucum. Phytopathology 90: 1169–1172 18944482 10.1094/PHYTO.2000.90.10.1169

[kiab040-B175] Wilson J , Hess D, Hanna W, Kumar K, Gupta S (2004) Pennisetum glaucum subsp. monodii accessions with *Striga* resistance in West Africa. Crop Prot 23: 865–870

[kiab040-B176] Xiang HB , Yao RF, Quan TF, Wang F, Chen L, Du XX, Zhang WH, Deng HT, Xie DX, Luo TP (2017) Simple beta-lactones are potent irreversible antagonists for strigolactone receptors. Cell Res 27: 1525–1528 28820177 10.1038/cr.2017.105 PMC5717398

[kiab040-B177] Xie XN , Yoneyama K, Yoneyama K (2010) The Strigolactone story. Annu Rev Phytopathol 48: 93–117 20687831 10.1146/annurev-phyto-073009-114453

[kiab040-B178] Yoneyama K , Awad AA, Xie X, Yoneyama K, Takeuchi Y (2010) Strigolactones as germination stimulants for root parasitic plants. Plant Cell Physiol 51: 1095–1103 20403809 10.1093/pcp/pcq055 PMC2900819

[kiab040-B179] Yoneyama K , Arakawa R, Ishimoto K, Kim HI, Kisugi T, Xie X, Nomura T, Kanampiu F, Yokota T, Ezawa T (2015) Difference in *Striga*‐susceptibility is reflected in strigolactone secretion profile, but not in compatibility and host preference in arbuscular mycorrhizal symbiosis in two maize cultivars. New Phytol 206: 983–989 25754513 10.1111/nph.13375

[kiab040-B180] Yoshida S , Shirasu K (2012) Plants that attack plants: molecular elucidation of plant parasitism. Curr Opin Plant Biol 15: 708–713 22898297 10.1016/j.pbi.2012.07.004

[kiab040-B181] Yoshida S , Cui S, Ichihashi Y, Shirasu K (2016) The haustorium, a specialized invasive organ in parasitic plants. Annu Rev Plant Biol 67: 643–667 27128469 10.1146/annurev-arplant-043015-111702

[kiab040-B182] Zarafi A , Elzein A, Abdulkadir D, Beed F, Akinola O (2015) Host range studies *of Fusarium oxysporum f. sp. strigae* meant for the biological control of *Striga hermonthica* on maize and sorghum. Arch Phytopathol Plant Protect 48: 1–9

[kiab040-B183] Zheng X , Giuliano G, Al-Babili S (2020) Carotenoid biofortification in crop plants: citius, altius, fortius. Biochimica et Biophysica Acta (BBA)-Molecular and Cell Biol Lipids 1865: 1–17 10.1016/j.bbalip.2020.158664 32068105

[kiab040-B184] Zimmermann J , Musyoki MK, Cadisch G, Rasche F (2016) Biocontrol agent *Fusarium oxysporum f.sp Strigae* has no adverse effect on indigenous total fungal communities and specific AMF taxa in contrasting maize rhizospheres. Fungal Ecol 23: 1–10 27721900 10.1016/j.funeco.2016.05.007 PMC5045157

[kiab040-B185] Zwanenburg B , Mwakaboko AS (2011) Strigolactone analogues and mimics derived from phthalimide, saccharine, p-tolylmalondialdehyde, benzoic and salicylic acid as scaffolds. Bioorg Med Chem 19: 7394–7400 22082666 10.1016/j.bmc.2011.10.057

[kiab040-B186] Zwanenburg B , Nayak SK, Charnikhova TV, Bouwmeester HJ (2013) New strigolactone mimics: structure-activity relationship and mode of action as germinating stimulants for parasitic weeds. Bioorg Med Chem Lett 23: 5182–5186 23920440 10.1016/j.bmcl.2013.07.004

[kiab040-B187] Zwanenburg B , Mwakaboko AS, Kannan C (2016) Suicidal germination for parasitic weed control. Pest Manag Sci 72: 2016–2025 26733056 10.1002/ps.4222

